# Prediction of Meniscal and Ligamentous Injuries in Lateral Tibial Plateau Fractures Based on Measurements of Lateral Plateau Widening on Multidetector Computed Tomography Scans

**DOI:** 10.1155/2018/5353820

**Published:** 2018-07-29

**Authors:** Jan P. Kolb, Marc Regier, Eik Vettorazzi, Norbert Stiel, Jan P. Petersen, Cyrus Behzadi, Johannes M. Rueger, Alexander S. Spiro

**Affiliations:** ^1^Department of Trauma, Hand, and Reconstructive Surgery, University Medical Center Hamburg-Eppendorf, Martinistrasse 52, 20246 Hamburg, Germany; ^2^Department of Diagnostic and Interventional Radiology, University Medical Center Hamburg-Eppendorf, Martinistrasse 52, 20246 Hamburg, Germany; ^3^Department of Medical Biometry and Epidemiology, University Medical Center Hamburg-Eppendorf, Martinistrasse 52, 20246 Hamburg, Germany; ^4^Department of Pediatric Orthopaedic Surgery, Children's Hospital, Hamburg-Altona, Germany; ^5^Department of Orthopaedics, University Medical Center Hamburg-Eppendorf, Hamburg, Germany

## Abstract

**Background:**

The influence of increasing lateral plateau widening on the frequency of meniscal and ligamentous lesions in lateral tibial plateau fractures has been examined in very few studies using plain radiographs. Because the amount of this parameter cannot be measured accurately on plain radiographs, the purpose of this survey was to look for a possible correlation between the extent of lateral plateau widening, as measured on multidetector CT (MDCT) scans, and different soft-tissue injuries determined from magnetic resonance imaging (MRI).

**Materials and Methods:**

55 patients with a lateral tibial plateau fracture were included in this retrospective case series. Patient age averaged 52.6 years (SD = 18.0). The degree of lateral plateau widening was measured on CT images. MRIs were screened for meniscal and ligamentous injuries.

**Results:**

We found a significant effect of increasing lateral plateau widening on the incidence of lateral meniscus lesions (P = 0.021), lateral collateral ligament tears (P = 0.047), and the overall quantity of meniscal and ligamentous lesions (P = 0.001).

**Discussion:**

MRIs are not widely used as a diagnostic tool in lateral plateau fractures of the tibia. Reasons might be the costs and the fact that it is a time-consuming examination. The results of this study may help to estimate the probability of specific soft-tissue lesions in lateral tibial plateau fractures based on measurements of lateral plateau widening on MDCT scans, and they may guide the decision for additional MRI and/or arthroscopically assisted repair.

## 1. Introduction

Clinical diagnosis of associated soft-tissue injuries in acute fractures of the tibial plateau is challenging, because of pain, swelling, and limited range of motion of the affected joint [1]. Arthroscopy has the advantage of direct visualization of soft-tissue lesions in the knee; however it is rarely used in the operative management of acute tibial plateau fractures. Magnetic resonance imaging is the favourable examination method in order to detect specific ligamentous and meniscal lesions in the knee, but it is not widely used in patients with fractures of the tibial plateau [2–5]. This may be due to the high cost, large amount of work, and limited availability of MRI [2–5].

Plain radiographs are usually taken to detect fractures of the tibial plateau. Computed tomography is used to determine the fracture type and to define a treatment plan, but neither plain radiographs nor CT give sufficient information about meniscal and ligamentous lesions [6–8]. Using MRI or intraoperative findings, previous studies have demonstrated that associated soft-tissue injuries are common in fractures of the tibial plateau, but there is a lack of studies reporting on the impact of lateral plateau widening on the frequency of specific soft-tissue lesions [1–5, 8–14].

In a former study we analyzed the effect of increasing tibial plateau depression, as measured on CT scans, on the frequency of meniscal and ligamentous lesions in patients with different fractures of the tibial plateau (Schatzker types I-VI), while the amount of lateral plateau widening was not assessed [5]. Increased tibial plateau depression had a significant effect on the incidence of anterior cruciate ligament tears and lateral meniscus lesions in this study [5].

In pure lateral tibial plateau fractures, the most common fracture types, measurements of lateral plateau widening and articular depression were performed in very few studies so far in order to predict soft-tissue lesions in the knee [9, 10]. Disadvantages of these studies are that the measurements were taken on plain radiographs [9, 10]. As described before we suggest that the degree of lateral plateau widening and articular depression cannot be measured precisely on plain radiographs [5, 10, 15]. Therefore, measurements of lateral plateau widening were performed on MDCT scans and they were correlated with different meniscal and ligamentous injuries determined from magnetic resonance images in order to evaluate the incidence rate of these lesions in lateral tibial plateau fractures.

## 2. Materials and Methods

Fifty-five patients with a fracture of the lateral tibial plateau were enclosed in this study. They presented to our emergency unit immediately after injury. Plain radiographs were taken at the day of injury. CT and MRI examination was performed at an average of 0.88 days (SD = 2.67) and 2.42 days (SD = 2.11) after trauma, respectively. MDCT was used to assess the fracture type and to define a treatment plan; MRIs (1,5T or 3T scanner) were screened for meniscal and ligamentous injuries, as described previously [5].

CT images were assessed by a trauma surgeon and a senior musculoskeletal radiologist (consensus decision). The fracture type was determined in each case according to the Schatzker classification. Only patients with a fracture of the lateral tibial plateau (Schatzker type I to III) were included in this study: split fracture (I; n=4); split-depression fracture (II; n=50); pure depression fracture (III; n=1) [2]. Lateral plateau widening and articular depression were measured on coronal CT scans. Lateral plateau widening was assessed as already described by Durakbasa et al. between a tangential line to the lateral femoral epicondyle and a parallel line from the most lateral point of the lateral tibial plateau, which were perpendicular to a line drawn from the extension of the medial plateau parallel to the joint line [9]. Articular depression was determined as described before [5].

A senior musculoskeletal radiologist assessed all MRI scans. Only grade 3 (linear elevated signal extending into the articular surface) and grade 4 (fragmented meniscal tear) lesions were considered to be meniscal tears in this series [16]. Disruption of up to 50% of the ligamentous fibres of the cruciate ligaments, the collateral ligaments, or the retinacula was referred to as a partial rupture [5]. A complete rupture was defined as a discontinuity of the ligaments [17]. Cruciate ligament footprint avulsion was evaluated as well.

### 2.1. Statistical Analysis

IBM SPSS (V. 19; Chicago, IL, USA) and R 2.13 (R Development Core Team, Vienna, Austria) was used. Mean and standard deviation (SD) or percentage was calculated. Logistic regression analyses were performed to evaluate the effect of increasing lateral plateau widening on each meniscal and ligamentous lesion. The correlation of lateral plateau widening, age, and gender with the overall quantity of meniscal and ligamentous lesions was analyzed by Pearson correlation coefficient. For all tests, significance was considered P < 0.05.

## 3. Results

55 patients with an acute fracture of the lateral tibial plateau were included in this retrospective study. 65.4% of the patients suffered from high energy trauma, including car (20.6%), motorcycle (17.7%), or kite surfing (8.8%) accidents. Patient age averaged 52.6 years (SD = 18.0). The left knee was involved in 53% and the right knee in 47% of cases, yielding a left to right side ratio of 1.1:1. The study cohort consisted of 67% female and 33% male patients, with a female to male ratio of 2.1:1. Lateral plateau widening was a mean of 4.4 mm (SD = 2.5); articular depression averaged 5.5 mm (SD = 5.1). Pearson correlation analysis revealed no correlation between the amount of depression and patient age. There was also no correlation between lateral plateau widening and patient age as calculated by Pearson correlation analysis.

Meniscal and ligamentous lesions were found in 78% of the patients; 43.6% had meniscal lesions (34.5% lateral; 20% medial); 58.2% had collateral ligament lesions (32.7% complete lateral collateral ligament lesion; 14.5% partial lateral collateral ligament lesion; 3.6% had complete medial collateral ligament lesion; 10.9% partial medial collateral ligament lesion); 23.6% had patellar retinaculum lesions (7.3% complete lateral patellar retinaculum lesion; 5.5% partial lateral patellar retinaculum lesion; 3.6% complete medial patellar retinaculum lesion; 9.1% partial medial patellar retinaculum lesion); 32.7% had cruciate ligament lesions (5.5% complete anterior cruciate ligament lesion; 23.6% partial anterior cruciate ligament lesion; 7.3% partial posterior cruciate ligament lesion; 1.8% posterior cruciate ligament footprint avulsion). Multiple soft-tissue injuries were detected in 50.9% of the patients.

**Figure 1:**
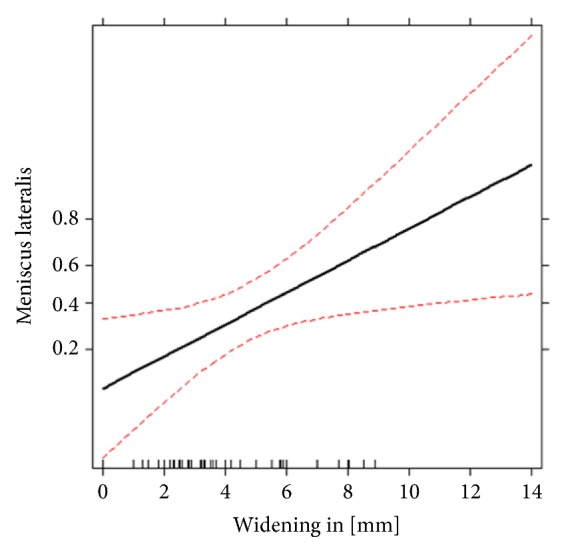
Effect of lateral plateau widening on the probability of meniscus lateralis tears estimated by logistic regression. Red lines are the estimated confidence limits. The y-axis denotes the estimated probability from the logistic regressions of having the given injury on logit scale.

**Figure 2:**
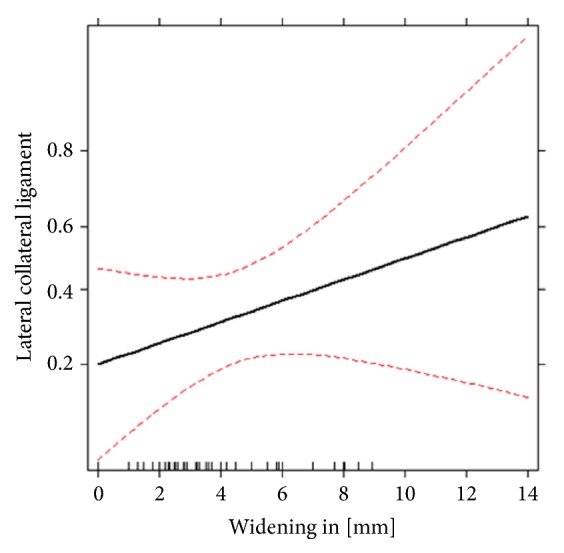
Effect of lateral plateau widening on the probability of lateral collateral ligament lesions estimated by logistic regression. Red lines are the estimated confidence limits. The y-axis denotes the estimated probability from the logistic regressions of having the given injury on logit scale.

**Figure 3:**
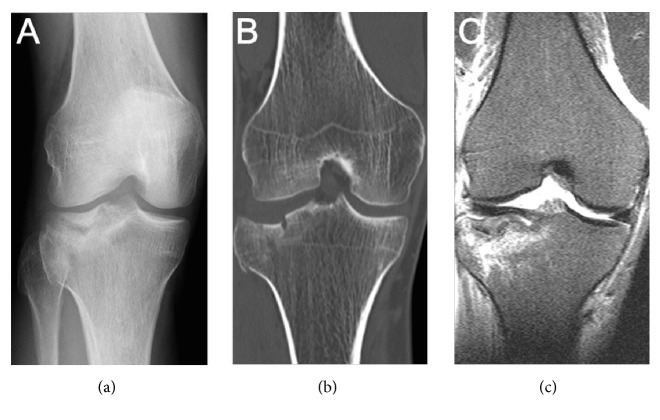
A 33.6-year-old female presented with a right lateral tibial plateau fracture. (a) Anteroposterior radiograph of the injured knee, (b) CT demonstrated a Schatzker II fracture with a maximum lateral plateau widening of 9 mm (coronal CT scan), and (c) MRI with a lateral collateral ligament lesion.

Logistic regression analysis (adjusted for age and gender) demonstrated a significant effect of increasing lateral plateau widening on the incidence of lateral meniscus lesions (P = 0.021) and lateral collateral ligament tears (P = 0.047). Enhancement of lateral plateau widening by 1.0 mm increases the risk of meniscus lateralis lesions (odds ratio 1.40, 95% CI 1.05–1.87) and lateral collateral ligament tears (odds ratio 1.32, 95% CI 1.00–1.73) up to 40% and 32%, respectively (Figures 1–3).

**Figure 4:**
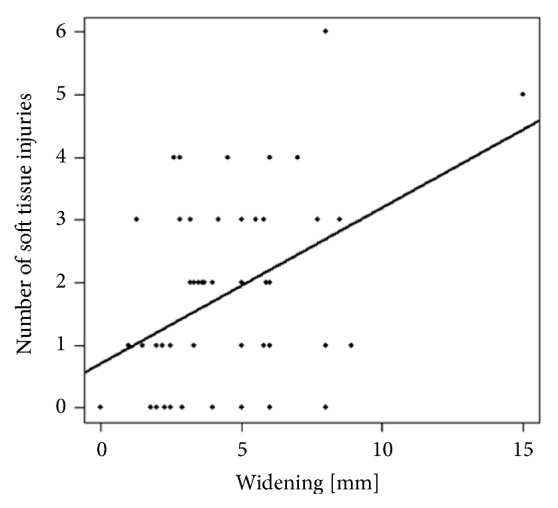
Scatter plot showing the absolute number of soft-tissue injuries versus lateral plateau widening, indicating a clear positive trend.

Pearson correlation analysis revealed a significant correlation between the extent of lateral plateau widening (P = 0.001), age (P = 0.007), and the overall quantity of meniscal and ligamentous lesions (Figure 4).

## 4. Discussion

Open reduction and internal fixation of lateral tibial plateau fractures may lead to poor outcome although anatomic realignment is achieved during surgery. This may be in part attributed to undetected meniscal and ligamentous injuries.

In this study, we found a significant effect of increasing lateral plateau widening on the incidence of specific soft-tissue injuries in lateral tibial plateau fractures. Our data indicate that lateral plateau widening, as measured on MDCT scans, may be used to predict the risk of lateral meniscus lesions and lateral collateral ligament tears in these fractures.

The influence of increasing lateral plateau widening on the frequency of meniscal and ligamentous lesions in lateral tibial plateau fractures has been examined in very few studies so far using plain radiographs [9, 10]. As described before, we believe that the extent of lateral plateau widening cannot be measured accurately on plain radiographs [5, 10, 15]. Therefore we used MDCT scans to evaluate this parameter and correlated the findings with different soft-tissue lesions determined from corresponding MR images in 55 patients with pure lateral tibial plateau fractures.

Durakbasa et al. assessed the amount of lateral plateau widening in a group of 20 patients with Schatzker type II fractures on plain radiographs and correlated these data with intraoperative findings [9]. Consistent with our results they could demonstrate that the amount of lateral plateau widening was greater in patients with a lateral meniscus lesion compared to those without such a lesion. However, none of the other meniscal and ligamentous lesions included in our study were analyzed in this small case series and the fact that patient age was not kept as adjustment variable in their statistical analysis may limit the validity of the author's conclusions.

Gardner et al. measured the amount of lateral plateau widening in 62 patients with Schatzker type II tibial plateau fractures using plain radiographs [10]. MR images were evaluated for soft-tissue injuries of the knee. The authors reported that lateral meniscus lesions occurred in 83% of the fractures with at least 5 mm of lateral plateau widening and 6 mm of plateau depression. However, in patients with displacement below these levels, lateral meniscus tears still had a 50% incidence rate. Their conclusion was that these results make clinical recommendations based on plain radiograph displacement less clear. Nevertheless, in accordance with the results of Gardner et al., we found a significant impact of increasing lateral plateau widening on the frequency of lateral meniscus lesions in lateral tibial plateau fractures. Furthermore we could demonstrate a significant correlation between the extent of lateral plateau widening and the frequency of lateral collateral ligament tears. These results are relevant in clinical practice, considering that the knowledge of specific meniscal and ligamentous tears may affect treatment and outcome of lateral tibial plateau fractures [2, 14, 18, 19].

The limitations of this retrospective study include the lack of randomization, the low number of patients, and the disadvantages of magnetic resonance imaging, as it may overdiagnose the extent of meniscal and ligamentous injuries in acute trauma patients. However, the utility of magnetic resonance imaging in diagnosing specific ligamentous and meniscal injuries in the knee has been well described in the literature [2–5, 10]. Meniscal lesions on MRI have been reported to occur in up to 25% of patients without trauma in this age group, which has to be considered when interpreting our results [10]. These lesions are classified as grade 1 to 4, but only grade 3 (linear elevated signal extending into the articular surface) and grade 4 (fragmented meniscal tear) lesions were considered to be meniscal tears in the present study [16]. Grade 1 (small focus of increased signal intensity that does not extend to the articular surface) and grade 2 (linear area of increased signal intensity that does not extend to the articular surface) lesions were not considered in our evaluation due to the fact that these lesions are frequently found in this age group as described [10, 16]. In addition, 34.5% of patients had a lateral meniscus lesion and only 20% had a medial meniscus lesion in this study, while in the majority of cases medial meniscus tears were evident in patients without trauma at this age [10]. We found a significant effect of increasing lateral plateau widening on the frequency of lateral meniscus lesions, but not on medial meniscus lesions in this series. Taken together, these data indicate that lateral meniscus tears may be attributed to the injury causing lateral tibial plateau fracture. Another limitation of this study is the wide age range. However, Pearson correlation analysis revealed no correlation between age and the extent of tibial plateau depression or lateral plateau widening. Thus, higher patient age along with osteoporosis had no bearing on depression and widening in this series. There was also no significant correlation between patient age and any specific soft-tissue lesion in the present study. Patient age was kept as adjustment variable in all analyses even if it was not significant. Therefore all reported effects of lateral plateau widening were adjusted for age and gender in this series.

Twenty-two patients were part of a previous analysis in which we assessed the impact of increasing articular depression only, but not lateral plateau widening, on the incidence of soft-tissue injuries in 54 different tibial plateau fractures [5]. Schatzker type I-VI fractures (fractures of the lateral and medial tibial plateau) were evaluated in our previous analysis compared to pure lateral tibial plateau fractures (Schatzker type I-III) in this study. Articular depression was also measured in the present series (statistical data not shown), but we could demonstrate that lateral plateau widening is superior to articular depression in predicting meniscal and ligamentous lesions in pure lateral tibial plateau fractures.

In conclusion, we found a significant effect of increasing lateral plateau widening on the incidence of lateral meniscus lesions, lateral collateral ligament tears, and the overall quantity of meniscal and ligamentous lesions in lateral tibial plateau fractures.

The high percentage of soft-tissue injuries, especially the high number of lateral collateral ligament injuries, may be explained by the high percentage of high energy trauma in this study. Due to the fact that MRI scans are associated with high costs and access is limited, the results of this study may help to estimate the probability of specific soft-tissue injuries in pure lateral tibial plateau fractures based on measurements of lateral plateau widening on MDCT scans. The determination of a cutoff in lateral plateau widening, where MRI or arthroscopic management should be definitely considered is not recommended by the authors. Soft-tissue lesions were also found in few patients with less displacement of lateral tibial plateau fracture in this study and in another prospective case series soft-tissue lesions were evident in non-displaced tibial plateau fractures as well [1]. Based on the results of this retrospective study, we recommend MRI in all patients with lateral tibial plateau fracture if available. However, prospective studies with a higher number of patients are needed to verify the findings of this study. Arthroscopic or open management of soft-tissue lesions during surgery should be considered if soft-tissue lesions were found on MRI.
